# Serum Parathyroid Hormone and Vitamin D Levels as Predictors of Hypocalcemia after Total/ Near Total Thyroidectomy

**DOI:** 10.1007/s12070-023-03599-3

**Published:** 2023-03-03

**Authors:** Priya Dugani, Poorvi V Sharma, Sunil M Krishna, Krishna Kalyan Reddy

**Affiliations:** 1 Department of General Surgery, Kasturba medical college and Hospital, Manipal Academy of Higher Education, Manipal, India; 2 Department of ENT, Kasturba Medical College, Manipal Academy of Higher Education, Manipal, India; 3 Department of General surgery, Kasturba Medical College, Manipal Academy of Higher Education, Manipal, India; 4 Durgabai Deshmukh Hospital and Research centre, Hyderabad, India

**Keywords:** Parathyroid hormone, Hypocalcaemia, Vitamin D, Total thyroidectomy, Near total thyroidectomy

## Abstract

Background: Post-operative Hypocalcaemia is the most-common complication of total and near-total thyroidectomy which is a selective treatment for benign and malignant thyroid diseases. Incidence ranges from 0.5-50%. Objectives: The role of vitamin-D and Parathyroid hormone(PTH) in incidence of Hypocalcemia after thyroidectomy has been taken into consideration. Methods: This is a prospective interventional study is conducted in Kasturba Medical College and hospital, Manipal after taking written informed consent from the participants. It aimed at surveying the serum level of preoperative Vitamin D, PTH and calcium before total-thyroidectomy surgery and its relationship with the incidence of postoperative hypocalcemia after the surgery. The study was done on 70 patients who were-planned for total/near total thyroidectomy. Preoperative Vitamin D, PTH, calcium and Postoperative 4 hours-PTH, Calcium were measured on POD-1, POD-2-4, the results obtained were then analysed. Results: Considering the cut-off of calcium as 8.6mg/dl, 42 patients developed hypocalcemia on POD-1, 28 patients on POD-2. Preoperative calcium and postoperative PTH levels in people having hypocalcaemia where significantly less compared to the patients having normal calcium. 4-hours post-operative PTH measurements showed 51% sensitivity, 100% specificity and strong co-relation between postoperative hypocalcemia and drop in PTH levels- (p=<0.001). Out of 42 patients who developed hypocalcemia 28- (65%) patients had vitamin-D deficiency(p=0.5) on POD-1 and out of 51 patients with hypocalcemia on POD 2-4, 33(78%) had-vitamin-D-deficiency(p=0.3852). Which was not statically significant. Conclusion:4 hours post-operative PTH level is a predictor of early postoperative hypocalcemia, by detecting this we can effectively manage postoperative hypocalcemia

## Introduction

Due to the increased number of thyroid malignancies, the patients undergoing total thyroidectomies has also increased. Post operative Hypocalcemia have also increased as it is the most common complication of total and near total thyroidectomy. Incidence ranges from 0.5 to 50% in post-operative patients [1]. Which occurs approximately 24–48 h post total or near total thyroidectomy surgeries.

Patients may have post op- paraesthesia, tingling, carpopedal spasm, cramps and numbness of extremities and perioral area. Chvostek sign, Trousseau sign, laryngospasm due to sustained muscle contraction, confusion, irritability and if prolonged may present with seizures. Prolonged QT interval may lead to torsades de pointes ventricular fibrillation [2].

The parathyroid glands are very fragile and supplied by Inferior thyroid artery. The short half-life of parathyroid hormone is 3–5 min which easily predisposes patients to hypocalcemia. Hypocalcemia may occur due to surgical trauma, devascularization, unintentional removal of parathyroid glands, reoperation. Even after meticulously performed procedures, some temporary parathyroid dysfunction may occur. Surgical excision/manipulation/ disruption of blood supply has been seen as a risk factor. The recommended surgical strategy is meticulous dissection and preservation of the parathyroid glands and their blood supply. Risk of complication is higher when fewer than three glands are identified during surgery. Depending upon the extent of parathyroid damage, postoperative hypocalcemia may be transient, which may resolve within a few months, or permanent, requiring lifelong oral or intravenous calcium supplementation [3, 4].

Vitamin D deficiency increases gut calcium absorption and also stimulates synthesis of PTH to maintain normal serum calcium levels [5]. Studies have shown that association between postoperative hypocalcemia and vitamin D deficiency leads to a longer hospital stay.

Definite framework in terms of post operative early identification of patients at risk of hypocalcaemia would allow for prophylactic treatment hence avoiding development of symptomatic hypocalcaemia and need for prolonged hospital stay.

Therefore, in our prospective interventional study, we have investigated the association between postoperative 4 h PTH and vitamin D with postoperative symptomatic and biochemical hypocalcemia in patients undergoing total and near total thyroidectomy.

## Methods

This was a single centred prospective interventional study conducted in the Department of General Surgery, surgical oncology and ENT at Kasturba Medical College and Hospital, Manipal between March 2021 to November 2022 after obtaining institutional ethics committee approval and written consent form all the 70 research participants. This study was sponsored by Roche Diagnostic India Pvt Ltd.

Patients above the age of 18 yrs who were planned for total and near total thyroidectomy surgeries were included in the study.

We excluded the patients who were on Calcium or Vitamin D supplementation, pre-excisting parathyroid disorders, hypocalcemia, paraneoplastic syndromes, chronic kidney disease or patients who had undergone previous neck dissections, thyroid surgeries, radiation to the neck, or auto-transplant of parathyroid gland.

Relevant preoperative details such as clinical, radiological, biochemical and pathological investigations. Pre-operative PTH, vitamin D and serum calcium were measured. After total or near total thyroidectomy at an interval of 4 hour, PTH was repeated, and POD 1, and 48 to 72 hour later serum calcium was repeated which was the standard of care. Signs and symptoms of hypocalcemia were observed. The patients were followed up till discharge and upto 3 months when the patient came for regular follow up to the OPD with serum calcium.

Data was coded and recorded in MS Excel spreadsheet program. SPSS v23 (IBM Corp.) and was used for data analysis.

## Results

The study population included 70 patients with the average age of 46.03 ± 13.41. Ten patients (14.3%) were males and 60 (85.7%) were females. 67 (95.7%) patients had neck swelling. 2 patients had hoarseness of voice, one patient had dysphagia with stridor and another patient had shortness of breath preoperatively. 69(patients where euthyroid (98.6%) and 1 patient had hypothyroidism. 27 (38.6%) patients had TIRADS 2 category on preoperative USG and 25 (35.7%) patients had TIRADS 4 category. 32 (47.8%) patients had FNAC report of colloid goiter. 6 (8.6%) patients underwent near total thyroidectomy and 64 (91.4%) underwent total thyroidectomy. Parathyroid gland and inferior thyroid artery was preserved in all cases. These parameters were not found to be statically significant in predicting biochemical or symptomatic hypocalcemia (Tables 1 and 2).

**Table 1:** Biochemical hypocalcaemia and its association with various parameters

Parameters	Yes (n = 51)	p value
**Age (Years)**	47.27 ± 13.69	0.262^1^
**Age**		0.899^2^
21–30 Years	4 (7.8%)	
31–40 Years	15 (29.4%)	
41–50 Years	13 (25.5%)	
51–60 Years	7 (13.7%)	
61–70 Years	9 (17.6%)	
71–80 Years	3 (5.9%)	
**Gender**		0.713^2^
Male	7 (13.7%)	
Female	44 (86.3%)	
**Neck Swelling (Yes)**	48 (94.1%)	0.562^2^
**Pressure Symptoms**		0.711^2^
No	48 (94.1%)	
Horseness Of Voice	1 (2.0%)	
Dysphagia With Stridor	1 (2.0%)	
Shortness Of Breath	1 (2.0%)	
**TFT**		1.000^2^
Normal	50 (98.0%)	
Hypothyroidism	1 (2.0%)	
**USG Neck**		0.887^2^
Tirads 1	5 (9.8%)	
Tirads 2	21 (41.2%)	
Tirads 3	1 (2.0%)	
Tirads 4	17 (33.3%)	
Tirads 5	5 (9.8%)	
Thyroiditis	1 (2.0%)	
Solitary Thyroid Nodule	1 (2.0%)	
**FNAC: Colloid Goiter (Yes)**	21 (41.2%)	0.092^3^
**FNAC: Adenomatoid Nodule (Yes)**	4 (7.8%)	1.000^2^
**FNAC: Papillary Carcinoma (Yes)**	8 (15.7%)	0.186^2^
**FNAC: Nodular Goiter (Yes)**	2 (3.9%)	1.000^2^
**FNAC: Follicular Neoplasm (Yes)**	8 (15.7%)	0.477^2^
**FNAC: Thyroiditis (Yes)**	3 (5.9%)	0.568^2^
**FNAC: Others (Yes)**	6 (11.8%)	0.670^2^
**Procedure**		1.000^2^
Near Total Thyroidectomy	5 (9.8%)	
Total Thyroidectomy	46 (90.2%)	
**Parathyroid (Preserved)**	51 (100.0%)	1.000^3^
**Inferior Thyroid Artery (Preserved)**	51 (100.0%)	1.000^3^
**Vitamin D (ng/mL) (Pre-Operative)**	19.18 ± 9.09	0.216^4^
**Vitamin D (Pre-Operative)**		0.385^2^
<20 ng/mL	33 (64.7%)	
20–30 ng/mL	14 (27.5%)	
≥30 ng/mL	4 (7.8%)	
**PTH (pg/mL) (Pre-Operative)**	48.05 ± 20.03	0.848^4^
PTH (pg/mL) (Post-Operative 4 h)***	19.00 ± 16.02	0.001^4^
**PTH (Pre-Operative)**		0.715^2^
15–65 pg/mL	42 (82.4%)	
>65 pg/mL	9 (17.6%)	
PTH (Post-Operative 4 h)***		< 0.001^3^
<15 pg/mL	28 (54.9%)	
15–65 pg/mL	23 (45.1%)	
**Calcium (mg/dL) (Pre-Operative)**	9.04 ± 0.44	0.230^4^
Calcium (mg/dL) (POD 1)***	8.16 ± 0.57	< 0.001^1^
Calcium (mg/dL) (POD 2–4)***	7.78 ± 0.60	< 0.001^4^
**Calcium (mg/dL) (3 Months)**	8.99 ± 0.87	0.061^4^
**Biochemical Hypocalcemia (Pre-Operative) (Yes)**	8 (15.7%)	0.101^2^
Biochemical Hypocalcemia (POD 1) (Yes)***	42 (60.0%)	< 0.001^3^
Biochemical Hypocalcemia (POD 2–4) (Yes)***	51 (73.9%)	< 0.001^3^
**Biochemical Hypocalcemia (3 Months) (Yes)**	10 (22.7%)	0.098^2^
Symptomatic Hypocalcemia (Yes)***	25 (49.0%)	0.001^3^
**Post Operative Complications**		1.000^2^
No	49 (96.1%)	
Horseness Of Voice	2 (3.9%)	
**Post Operative Management: No (Yes)**	9 (17.6%)	0.102^2^
Post Operative Management: IV Calcium (Yes)***	18 (35.3%)	0.015^2^
**Post Operative Management: Oral Calcium (Yes)**	41 (80.4%)	0.121^2^
**Post Operative Management: Vitamin D (Yes)**	14 (27.5%)	0.528^2^
**Discharged with Calcium Supplementation: No (Yes)**	9 (18.4%)	0.205^2^
**Discharged with oral Calcium and vitamin DSupplementation**	40 (81.6%)	0.205^2^
**Final HPE Impression**		1.000^3^
Benign	34 (66.7%)	
Malignant	17 (33.3%)	
Change in PTH (pg/mL) (Post-Operative 4 h)***	-29.05 ± 22.81	0.032^4^

**Table 2:** Symptomatic hypocalcaemia and its association with various parameters

Parameters	Yes (n = 26)	P value
**Age (Years)**	44.65 ± 13.74	0.518^1^
**Age**		0.682^2^
21–30 Years	4 (15.4%)	
31–40 Years	8 (30.8%)	
41–50 Years	6 (23.1%)	
51–60 Years	3 (11.5%)	
61–70 Years	3 (11.5%)	
71–80 Years	2 (7.7%)	
**Gender**		0.734^2^
Male	3 (11.5%)	
Female	23 (88.5%)	
**Neck Swelling (Yes)**	25 (96.2%)	1.000^2^
**Pressure Symptoms**		0.441^2^
No	25 (96.2%)	
Horseness Of Voice	0 (0.0%)	
Dysphagia With Stridor	0 (0.0%)	
Shortness Of Breath	1 (3.8%)	
**TFT**		1.000^2^
Normal	26 (100.0%)	
Hypothyroidism	0 (0.0%)	
**USG Neck**		0.775^2^
Tirads 1	3 (11.5%)	
Tirads 2	13 (50.0%)	
Tirads 3	0 (0.0%)	
Tirads 4	9 (34.6%)	
Tirads 5	1 (3.8%)	
Thyroiditis	0 (0.0%)	
Solitary Thyroid Nodule	0 (0.0%)	
**FNAC: Colloid Goiter (Yes)**	10 (38.5%)	0.226^3^
**FNAC: Adenomatoid Nodule (Yes)**	0 (0.0%)	0.149^2^
**FNAC: Papillary Carcinoma (Yes)**	5 (19.2%)	0.143^2^
**FNAC: Nodular Goiter (Yes)**	2 (7.7%)	0.552^2^
**FNAC: Follicular Neoplasm (Yes)**	5 (19.2%)	0.754^2^
**FNAC: Thyroiditis (Yes)**	1 (3.8%)	1.000^2^
**FNAC: Others (Yes)**	3 (11.5%)	1.000^2^
**Procedure**		0.078^2^
Near Total Thyroidectomy	0 (0.0%)	
Total Thyroidectomy	26 (100.0%)	
**Parathyroid (Preserved)**	26 (100.0%)	1.000^3^
**Inferior Thyroid Artery (Preserved)**	26 (100.0%)	1.000^3^
**Vitamin D (ng/mL) (Pre-Operative)**	20.18 ± 9.53	0.771^4^
**Vitamin D (Pre-Operative)**		0.537^2^
<20 ng/mL	15 (57.7%)	
20–30 ng/mL	8 (30.8%)	
≥30 ng/mL	3 (11.5%)	
**PTH (pg/mL) (Pre-Operative)**	47.46 ± 22.85	0.789^4^
PTH (pg/mL) (Post-Operative 4 h)***	11.45 ± 11.20	< 0.001^4^
**PTH (Pre-Operative)**		1.000^2^
15–65 pg/mL	22 (84.6%)	
>65 pg/mL	4 (15.4%)	
PTH (Post-Operative 4 h)***		< 0.001^3^
<15 pg/mL	20 (76.9%)	
15–65 pg/mL	6 (23.1%)	
**Calcium (mg/dL) (Pre-Operative)**	9.03 ± 0.40	0.137^4^
Calcium (mg/dL) (POD 1)***	7.93 ± 0.58	< 0.001^1^
Calcium (mg/dL) (POD 2–4)***	7.48 ± 0.64	< 0.001^1^
Calcium (mg/dL) (3 Months)***	8.79 ± 1.05	0.038^4^
**Biochemical Hypocalcemia (Pre-Operative) (Yes)**	3 (11.5%)	1.000^2^
Biochemical Hypocalcemia (POD 1) (Yes)***	22 (84.6%)	0.001^3^
Biochemical Hypocalcemia (POD 2–4) (Yes)***	25 (96.2%)	0.001^3^
Biochemical Hypocalcemia (3 Months) (Yes)***	7 (30.4%)	0.037^2^
Biochemical Hypocalcemia (Yes)***	25 (100.0%)	0.001^3^
**Post Operative Complications**		0.289^2^
No	26 (100.0%)	
Horseness Of Voice	0 (0.0%)	
Post Operative Management: No (Yes)***	1 (3.8%)	0.002^3^
Post Operative Management: IV Calcium (Yes)***	17 (65.4%)	< 0.001^3^
Post Operative Management: Oral Calcium (Yes)***	25 (96.2%)	0.001^3^
Post Operative Management: Vitamin D (Yes)***	13 (50.0%)	< 0.001^3^
Discharged Oral Calcium and vitamin D supplementation(Yes)***	24 (96.0%)	0.004^3^
**Final HPE Impression**		0.775^3^
Benign	18 (69.2%)	
Malignant	8 (30.8%)	
Change in PTH (pg/mL) (Post-Operative 4 h)***	-36.01 ± 22.89	0.001^4^

Mean preoperative calcium, PTH and vitamin D and PTH 4 h post operative, and calcium on POD 1, 2–4 days after surgery have been presented in the Table 3. Preoperative PTH was found to be within normal range in 59 (84.3%) patients and elevated in 11 (15.7%), and post operative 4 h, 42 (60%) had PTH between 15 and 65 pg/mL (normal range) and 28 (40%) patients had low levels of PTH(< 15 pg/mL).

**Table 3:** Mean pre and postoperative PTH and calcium distribution

PTH	Mean ± SD, Frequency (%)
Pre-Operative	
15–65 pg/mL	59 (84.3%)
>65 pg/mL	11 (15.7%)
**Post-Operative 4 h**	
<15 pg/mL	28 (40.0%)
15–65 pg/mL	42 (60.0%)
**Calcium (mg/dL)**	**Mean ± SD**
**Pre-Operative**	9.07 ± 0.41
**POD 1**	8.33 ± 0.62
**POD 2–4**	8.08 ± 0.73
**3 Months**	9.11 ± 0.80

Preoperative Vitamin D deficiency was noted in 43 (61.4%) study population, 22 (31.4%) had vitamin D in the normal range and 5 (7.1%) patients had elevated levels of vitamin D as shown in Table 4.

**Table 4:** Frequency of vitamin D( preoperative)

Vitamin D (Pre-Operative)	Frequency
**< 20 ng/mL**	43 (61.4%)
**20–30 ng/mL**	22 (31.4%)
**≥ 30 ng/mL**	5 (7.1%)
**Hypocalcemia**	Yes
**Biochemical Hypocalcemia**	51 (73.9%)
**Symptomatic Hypocalcemia**	26 (37.1%)

On POD 1 serum calcium ranged from 8.33 ± 0.62 ml/dl and 8.08 ± 0.73 ml/dl on POD 2–4.

On POD 1, 42 (60.0%) patients, on POD 2–4, 51 (73.9%) patients developed biochemical hypocalcaemia. 10 (16.9%) patients had persistent hypocalcemia on 3 months follow up as depicted in Table 5.

**Table 5:** Population who developed post operative biochemical hypocalcemia

Biochemical Hypocalcemia	Yes
**POD 1**	42 (60.0%)
**POD 2–4**	51 (73.9%)
**3 Months**	10 (16.9%)

27 (49%) patients developed symptomatic hypocalcemia, of which 25 (49.0%) had biochemical hypocalcemia and 2 (5.6%) patients did not have biochemical hypocalcemia.

**Graph 1:**
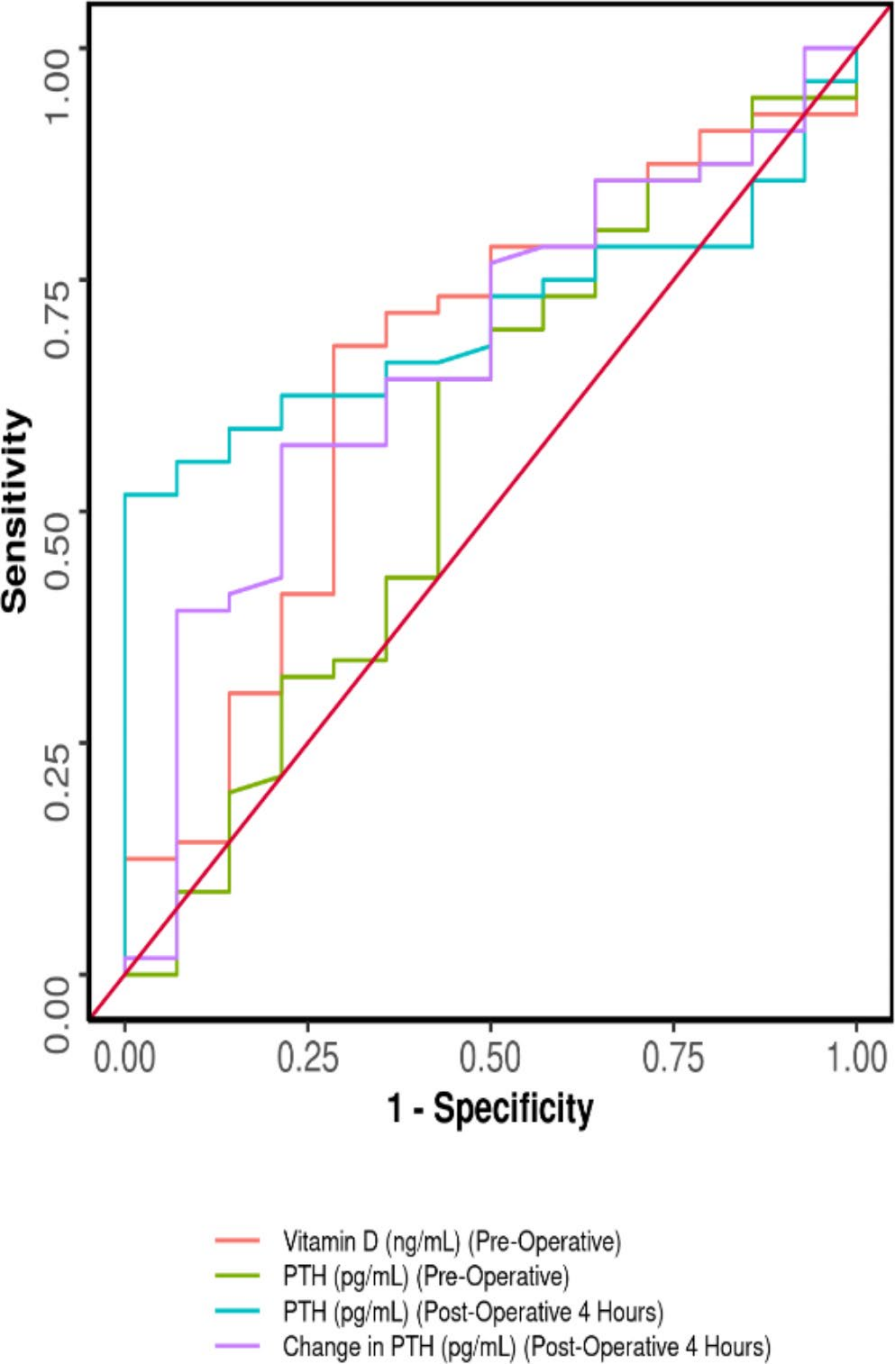
Depicting AUROC of various parameters in predicting biochemical hypocalcaemia

On POD 1, post operative 4 hour PTH was measured, 24 (85.7%) patients had low levels of PTH, 18 (42.9%) patients had normal PTH. 28 (100.0%) had low levels of PTH (< 15pg/ml) 23 (56.1%) had normal PTH developed biochemical hypocalcemia on POD 2–4. On 3 months follow up, 9(36%) patients who had low PTH and 1(2.9%) had persistent biochemical hypocalcemia. This was noted to be statically significant in predicting biochemical hypocalcemia (p = < 0.001). Change in PTH preoperatively and post operatively was also found to be just statically significant (p = 0.05) as shown in Graph 1.

On POD 1, 2–4, vitamin D was compared with calcium levels and was not found to be significant (P = 0.863, 0.932).

As described in Table 6, Specificity of post operative 4 h PTH and change in PTH was found to be 100% and 78.6% respectively, and PPV of 100% and 91.4% in predicting biochemical hypocalcemia.

**Table 6:** Post operative 4 h PTH change in PTH and preoperative Vitamin D as predictors of biochemical hypocalcemia

Parameter	4 h post operative PTH Value (95% CI)	Change in PTH Value (95% CI)	Vitamin D Value (95% CI)e (95% CI)
Cutoff (p value)	≤ **15.1 (0.017)**	≤ -22.7 **(0.05)**	≤ 19.49 (0.074)
AUROC	0.707 (0.589–0.826)	0.666 (0.508–0.823)	0.656 (0.488–0.823)
Sensitivity	51.8% (38–65)	57.1% (43–70)	67.9% (54–80)
Specificity	**100.0% (77–100)**	**78.6% (49–95)**	71.4% (42–92)
Positive Predictive Value	**100.0% (88–100)**	**91.4% (77–98)**	**90.5% (77–97**)
Negative Predictive Value	34.1% (20–51)	31.4% (17–49)	35.7% (19–56)
Diagnostic Accuracy	61.4% (49–73)	61.4% (49–73)	**68.6% (56–79)**
Positive Likelihood Ratio	Inf (NaN-Inf)	2.67 (0.95–7.46)	2.38 (1.02–5.54)
Negative Likelihood Ratio	0.48 (0.37–0.63)	0.55 (0.36–0.82)	0.45 (0.27–0.75)
Diagnostic Odds Ratio	Inf (NaN-Inf)	4.89 (1.23–19.47)	5.28 (1.46–19.14)

**Table 7:** Post operative 4 h PTH change in PTH and preoperative Vitamin D as predictors of symptomatic hypocalcemia as shown in Table 7 and Graph 2.

Parameter	Post operative 4 h PTH Value (95% CI)	Change in PTH (95% CI)	Vitamin D Value (95% CI)
Cutoff (p value)	≤ 9.1 (< 0.001)	≤ -13.3 (0.001)	≥ 25.06 (0.771)
AUROC	0.847 (0.742–0.952)	0.734 (0.612–0.855)	0.521 (0.371–0.672)
Sensitivity	76.9% (56–91)	92.3% (75–99)	34.6% (17–56)
Specificity	88.6% (75–96)	47.7% (32–63)	84.1% (70–93)
Positive Predictive Value	80.0% (59–93)	51.1% (36–66)	56.2% (30–80)
Negative Predictive Value	86.7% (73–95)	91.3% (72–99)	68.5% (54–80)
Diagnostic Accuracy	84.3% (74–92)	64.3% (52–75)	65.7% (53–77)
Positive Likelihood Ratio	6.77 (2.89–15.86)	1.77 (1.3–2.39)	2.18 (0.92–5.14)
Negative Likelihood Ratio	0.26 (0.13–0.53)	0.16 (0.04–0.63)	0.78 (0.57–1.06)
Diagnostic Odds Ratio	26 (7.06–95.74)	10.96 (2.3-52.09)	2.8 (0.89–8.77)

**Graph 2:**
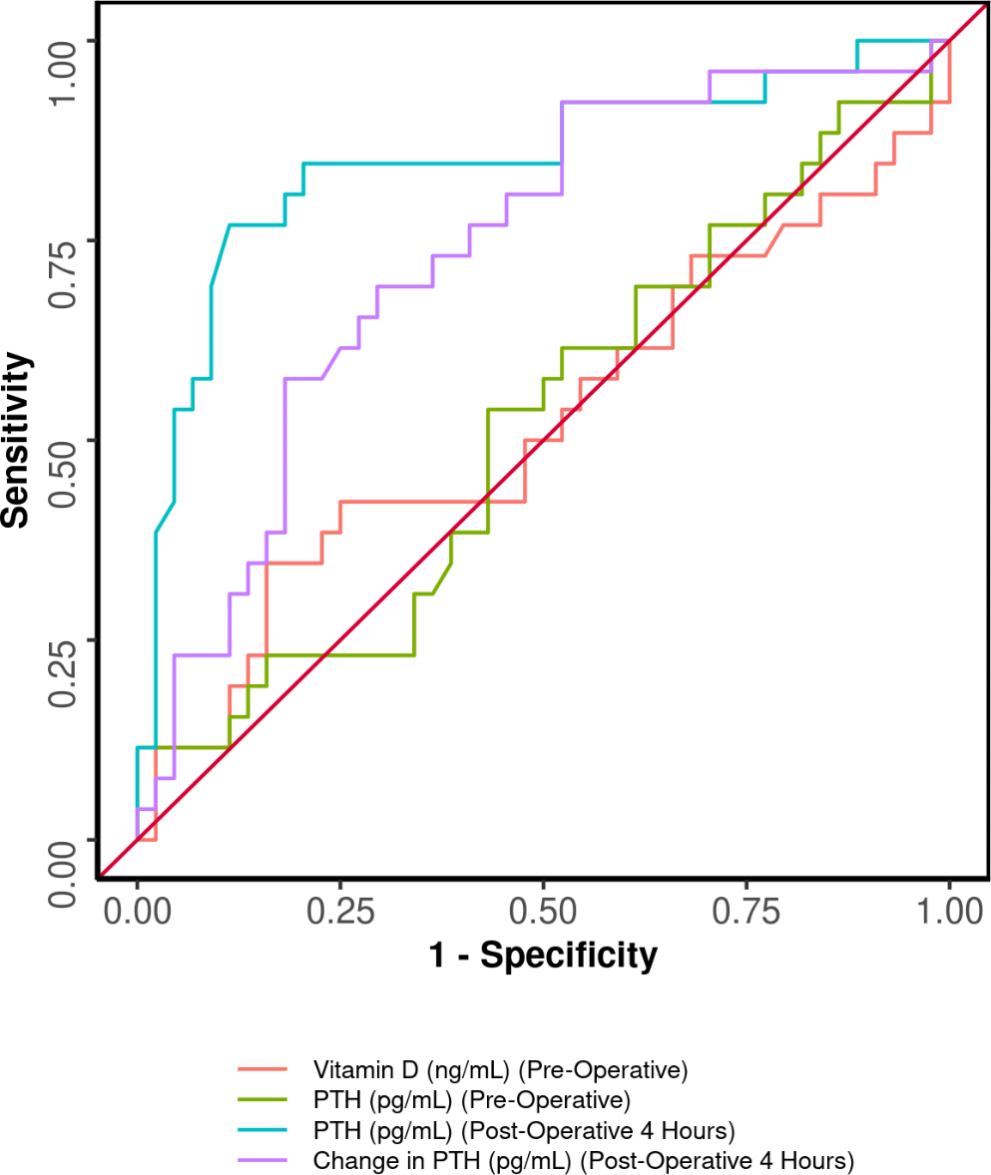
Depicting AUROC of various parameters in predicting symptomatic hypocalcemia

Vitamin D has high PPV of 90.5% and diagnostic accuracy of biochemical hypocalcemia of 68.6%.

Post operative 4 h PTH and Specificity of 88% and NPV of 86% vitamin D has 84% specificity in predicting symptomatic hypocalcemia. Change in post operative PTH has NPV of 91% in predicting symptomatic hypocalcemia.

47 patients had benign final histopathology and 23 patients and malignant, this did not provide any statical significance in predicting biochemical or symptomatic hypocalcemia (p = 0.775, 1 respectively). 19 (27%) patients required post operative IV calcium correction, 52(74%) patients required oral calcium and 17 (24%) patients required additional vitamin D correction. 52 (76.5%) patients were given oral vitamin D and calcium supplementation on discharge.

## Discussion

Total and near total thyroidectomy are routinely performed surgeries. Hypocalcemia is the complication which occurs 24–48 h after the surgery. This prolongs the hospital stay of the patient. Different investigators have used numerous parameters to predict the possibility of hypocalcemia post operatively.

In a study conducted by Noureldine et al. [6] 304 total thyroidectomies were performed, mild and significant hypocalcemia occurred in 22.4% and 29.9% patients respectively, 24% developed hypocalcemia-related symptoms. Mean age of patients who developed biochemical hypocalcemia was between 50.7 and 45.9 yrs (P = 0.02) with female preponderance (P = 0.003) which was statistically significant. 23.35% of the patients had preoperative diagnosis of Papillary neoplasm and 14.8% patients had goiter.

Guk Haeng Lee et al. [7] analysed 134 patients who underwent Total thyroidectomy for thyroid malignancies and developed laboratory and symptomatic hypocalcemia in 39% and 19% participants, on the day after surgery respectively. The mean age of patients who developed laboratory hypocalcemia was 52 ± 14 yrs with 46 female patients and mean age of 48 ± 10 yrs and 23 female patients developing symptomatic hypocalcemia.

In our study the youngest patient was 22 yrs and oldest patient was 77 yrs with a mean age of 46.03 ± 13.41 yrs. 30.8% patients who developed symptomatic hypocalcemia and 33.9% patients who developed biochemical hypocalcemia where between the age of 31–40 yrs respectively.

12.5% males and 87.5% females developed biochemical hypocalcemia respectively and 11.5% and 88.5% females presented with symptomatic hypocalcemia.

Postoperative and intraoperative serum-PTH has been analysed in various studies to predict hypocalcemia in post-total thyroidectomy [8–10]. Noordzij et al. analysed 9 observational studies and found that 6 h post-operative PTH value in total thyroidectomies had sensitivity of 96.4% and specificity of 91.4% n detecting hypocalcemia postoperatively. Lombardi et al. measured PTH at 2, 4, 6, 24, and 48 h postoperatively and found that the best predictive value of hypocalcemia was given by PTH at 4 and 6 h. Payne et al. measured PTH 6, 12, 20 h postoperatively and concluded that 12 h PTH measurement was the most sensitive.

As there are no definitive guidelines for measuring postoperative PTH, we used 4 h post operative PTH as the predictor in our study. The cut-off of PTH is different in different studies [8–13]. In the review conducted by Noordzij et al. [10], mean PTH was found to be 13.52 pg/mL in patients who developed post operative hypocalcemia. Payne et al. [8] found that Serum PTH levels less than 10 pg/ml was best predictor of post operative hypocalcemia. Asari et al. [14] and Roh and Park [15] found that the best predictor of hyocalcemia is PTH less than or equal to 15 pg/mL. Ramalingam et al. [16] showed that the postoperative PTH measured at 8 h post operatively, measuring 10 pg/mL had sensitivity of 68% and specificity of 73%.

Indian patients were found to have vitamin D deficiency in various studies [14–16]. Preoperative vitamin-D is considered a predictor of hypocalcemia as it directly influences calcium metabolism [17, 18]. Lips et al. [19] has shown that low levels of vitamin D levels increased the chance of hypocalcemia by 28 times in patients who undergo total thyroidectomy. Ramalingam et al. [16] showed that vitamin D levels equal to or less than 20 ng/mL had sensitivity of 68% and specificity of 80% in predcting post operative hypocalcemia.

Abdollahi et al. [20] conducted a study where 35.1% patients had Vitamin D deficiency. 70% had postoperative Hypocalcemia on the first day and 86% on post operative day 2, but it was found not to be statically significant (P = 0.166).

In a study by Cherian et al. [21] Vitamin D deficiency was present in 53.3% participants, hypocalcemia was observed in 44.7% participants, of which there was similar distribution of hypocalcemia in patients with normal vitamin D and deficient groups(48.6% and 41.3%, respectively) P = 0.23, hence concluded that Vitamin D was not associated with hypocalcemia in post thyroidectomy patients.

In our study we analysed whether preoperative Vitamin D and 4 hour post operative PTH can reliably predict hypocalcemia in post operative period. 43 patients (61.4%) in our study had vitamin D deficiency (< 20ng/ml) which was significant. Vitamin-D deficiency however did not show any association with post operative hypocalcemia (P = 0.074). Vitamin D has 90.5% PPV and 71.4% specificity with a diagnostic accuracy of 68.6%.

The role of vitamin D in relation to thyroid and parathyroid is still being studied. Reports have shown that Vitamin D levels vary with stage of thyroid malignancy and autoimmune thyroiditis [22, 23]. Hence the possibility that vitamin D may be a marker for disease severity and not a predictor of hypocalcaemia.

In a study conducted by Lang et al. [24] out of the 17 patients who developed biochemical hypocalcemia in the post operative period, 9 patients had benign pathology(p = 0.028), 5 patients had grave’s disease and 3 patients had malignant final histopathology. Noureldine et al. [6] in his study with 304 patients 159 patients developed hypocalcemia of which 104 patients had malignant pathology which was found to be statistically significant. In our study, 34 patients who developed biochemical hypocalcemia had benign disease and 17 patients had malignant disease in the final histopathology which was not found to be statically significant.

## Conclusion

Parathyroid hormone which is measured at 4 hour post operatively (< 15 pg/mL) and change in PTH after total and near total thyroidectomy accurately predicts post-operative hypocalcemia. The early detection of patients who are at risk of developing post-operative hypocalcemia facilitates prompt supplementation of calcium and Vitamin D and reduces morbidity, life threatening complications and permits early discharge.
